# Decreased TCL6 expression is associated with poor prognosis in patients with clear cell renal cell carcinoma

**DOI:** 10.18632/oncotarget.11011

**Published:** 2016-08-02

**Authors:** Hengchuan Su, Tiantian Sun, Hongkai Wang, Guohai Shi, Hailiang Zhang, Fukang Sun, Dingwei Ye

**Affiliations:** ^1^ Department of Urology, Fudan University Shanghai Cancer Center, Shanghai, China; ^2^ Department of Oncology, Shanghai Medical College, Fudan University, Shanghai, China; ^3^ Division of Gastroenterology and Hepatology, Renji Hospital, School of Medicine, Shanghai Jiao Tong University, Shanghai Institute of Digestive Disease, Shanghai, China; ^4^ Department of Urology, Ruijin Hospital, School of Medicine, Shanghai Jiaotong University, Shanghai, China

**Keywords:** clear cell renal cell carcinoma, lncRNA, microarray analysis, TCL6, prognosis

## Abstract

One-third of clear cell renal cell carcinoma (ccRCC) patients present with metastasis at the time of diagnosis. The prognosis of these patients is poor. To identify potential prognostic biomarkers and therapeutic targets for ccRCC, we re-evaluated published long non-coding RNA (lncRNA) expression profiling data from the Gene Expression Omnibus and ArrayExpress database. We found that five lncRNAs were differentially expressed in ccRCC and adjacent tissues. These lncRNAs were assessed in an independent cohort of 71 paired patient samples using real-time PCR. Differences in expression of three of the lncRNAs (ENSG00000177133, TCL6, and ENSG00000244020) were validated in this analysis. Kaplan-Meier analysis indicated that low expression of ENSG00000177133 and TCL6 was associated with a poor prognosis. Univariate and multivariate regression analyses demonstrated that TCL6 but not ENSG00000177133 expression was an independent predictor of ccRCC aggressiveness and had hazard ratios predictive of clinical outcome. TCL6 expression was negatively correlated with pTNM stage. Overexpression of TCL6 in 786-O and Caki-1 ccRCC cells decreased proliferation and increased apoptosis compared to controls. Our results indicate that lncRNA expression is altered in ccRCC and that decreased TCL6 expression may be an independent adverse prognostic factor in ccRCC patients.

## INTRODUCTION

Renal cell carcinoma (RCC) is the third most common genitourinary cancer and accounts for approximately 3% of all cancers [1, 2]. Approximately 70% of all renal tumors are clear cell RCC (ccRCC) [3]. Despite the use of ultrasound and computed tomography, approximately one-third of ccRCC patients present with metastasis at the time of diagnosis, which is associated with a poor prognosis. The identification of sensitive and specific ccRCC biomarkers and the development of new therapeutic approaches is essential.

Microarrays and high-throughput RNA sequencing tools have facilitated the identification of long non-coding RNAs (lncRNAs) that modulate gene expression [4]. Previous studies have indicated that lncRNAs play critical roles in malignant tumors including colorectal and ovarian cancer [5, 6]. However, the functions of lncRNAs in ccRCC development and metastasis have not been elucidated [7].

In order to identify potential biomarkers for ccRCC, we analyzed previously published ccRCC microarray datasets and applied more stringent filtering criteria [8, 9]. We identified a series of differentially expressed lncRNAs in ccRCC, which were validated in clinical samples by real-time PCR (RT-PCR). Associations between these lncRNAs and ccRCC patient clinicopathological features and prognosis were then investigated. Univariate and multivariate regression analyses were then used to determine whether the expression of each lncRNA was an independent predictor of ccRCC aggressiveness. Finally, the impact of overexpression or knockdown of each lncRNA on cell proliferation and apoptosis was evaluated. The expression profiles of these lncRNAs may be beneficial for the early diagnosis and treatment of ccRCC.

## RESULTS

### Patient clinical characteristics

A total of 71 patients were enrolled in the study. The detailed clinicopathological data for the patients are shown in Table 1. The median age was 55 years. There were 52 men and 19 women. Of these patients, 49 had histological stage I–II and 22 had histological stage III–IV disease. There were 27 patients with Fuhrman grade I–II and 44 with Fuhrman grade III–IV ccRCC. Follow-up data was available for 71 patients. The median observation period was 53 months (range: 2–79 months). There were 54 patients who were alive at the last clinical follow-up. Finally, there were 17 cancer-related deaths at the time of the last follow-up.

**Table 1:** Clinicopathological features of 71 patients with ccRCC

Clinicopathological features	*N* (%) of patients
**Median Age (years)**	
< 55	36 (50.7%)
≥ 55	35 (49.3%)
**Gender**	
Male	52 (73.2%)
Female	19 (26.8%)
**Tumor location**	
Left	35 (49.3%)
Right	36 (50.7%)
**TNM stage**	
Stage I	35 (49.3%)
Stage II	14 (19.7%)
Stage III	13 (18.3%)
Stage IV	9 (12.7%)
**T classification**	
T1	36 (50.7%)
T2	17 (24.0%)
T3	14 (19.7%)
T4	4 (5.6%)
**Lymph node metastasis**	
Negative	64 (90.1%)
Positive	7 (9.9%)
**Distant metastasis**	
Negative	65 (91.5%)
Positive	6 (8.5%)
**Fuhrman Grade**	
I	2 (2.8%)
II	25 (35.2%)
III	36 (50.7%)
IV	8 (11.3%)
**Survival status**	
Median OS (months)	53
Alive	54 (76.1%)
Dead	17 (23.9%)

### Dataset characteristics

**Figure 1:**
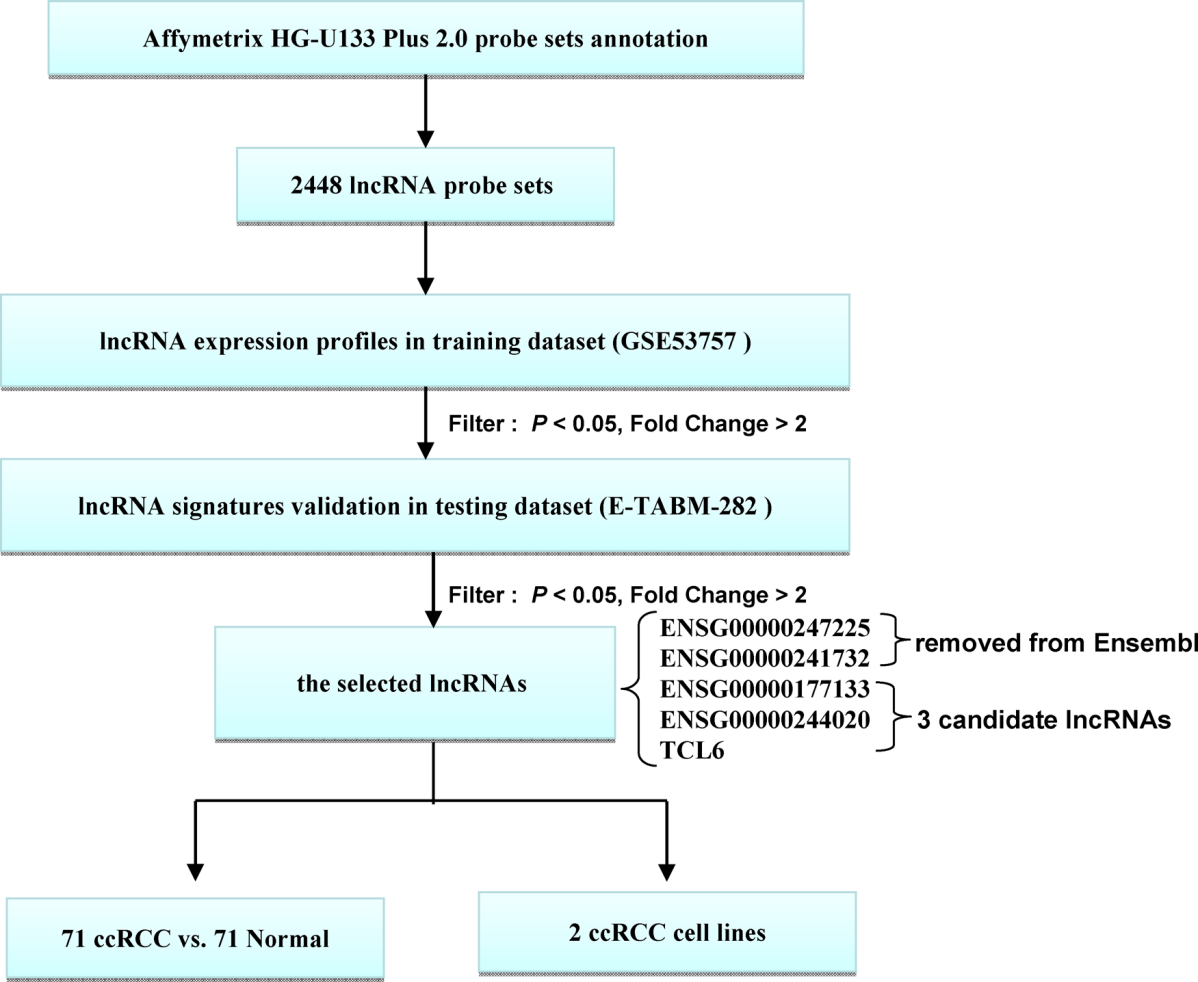
Schematic of the workflow All analyses were performed with the GSE53757 and E-TABM-282 datasets.

There were 72 ccRCC samples in the GSE-53757 dataset: 24 stage I, 19 stage II, 14 stage III, and 15 stage IV samples. This dataset also contained 72 adjacent normal tissue samples. The E-TABM-282 dataset contained 16 ccRCC samples: eight stage I, five stage II, three stage III, and no stage IV samples. This dataset also contained 11 adjacent normal tissue samples. GSE-53757 served as the training dataset from which gene expression signatures were derived, and E-TABM-282 served as the test dataset in which the results were confirmed. A schematic of the workflow for the entire study is shown in Figure 1.

### Microarray screening for differentially expressed lncRNAs in ccRCC tissue

**Figure 2:**
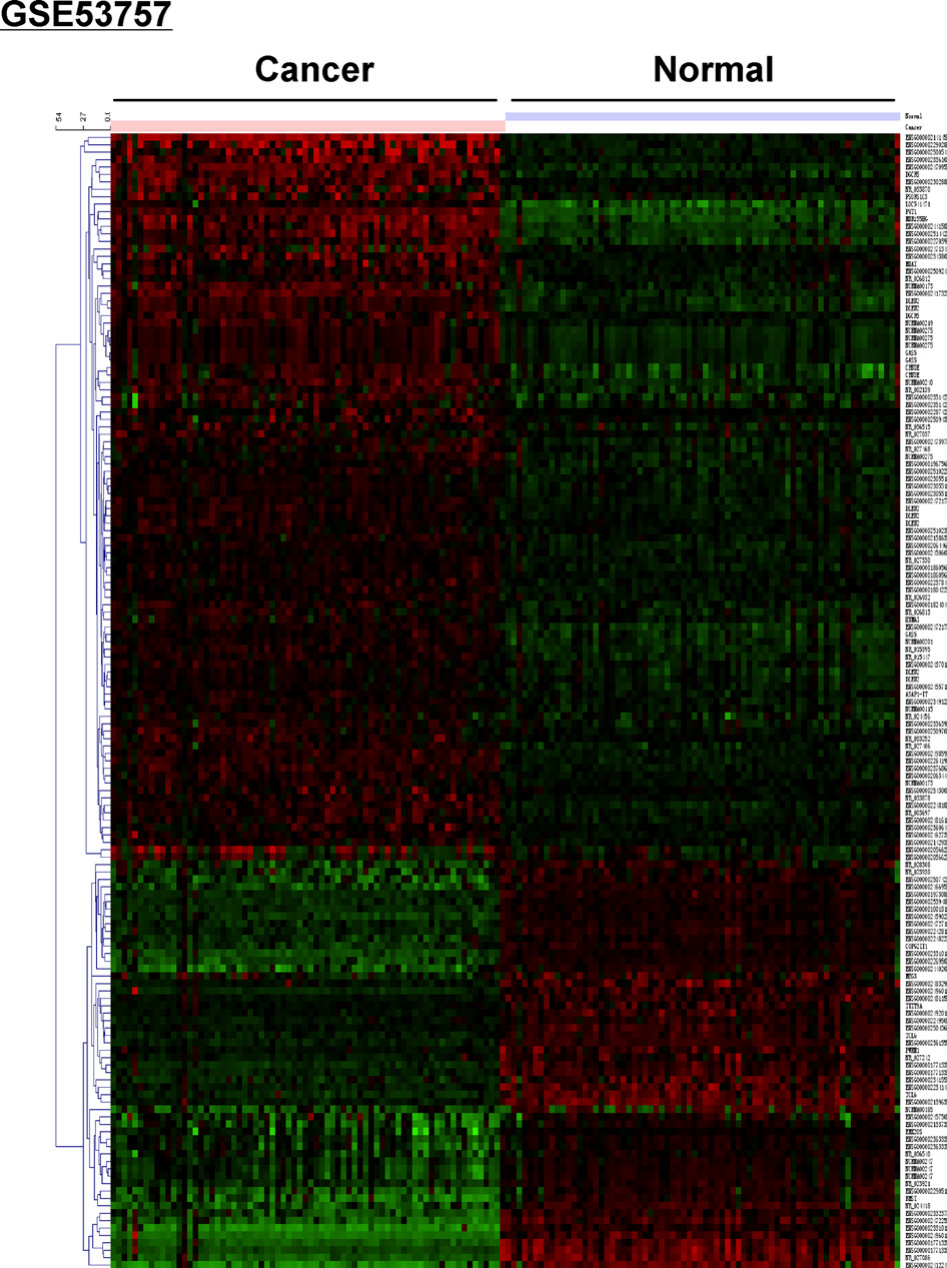
Microarray analysis showing differential expression of lncRNAs between ccRCC and normal renal tissue in the GSE53757 dataset The red and green colors indicate high and low expression, respectively. The GSE53757 dataset included 72 ccRCC and paired normal renal tissue samples.

We first used the GSE-53757 dataset as a discovery cohort in order to identify lncRNAs that were differentially expressed in 72 tumors and the corresponding normal renal tissue. Heat maps revealed that the expression profiles of 2,448 different lncRNA transcript variants allowed accurate discrimination between normal and malignant renal tissue (Figure 2). Of the 2,448 lncRNA transcripts analyzed, differential expression of 103 was observed (fold change > 2, *P* < 0.05). There were 48 lncRNAs that were upregulated and 55 downregulated in ccRCC samples. We next examined the expression of these lncRNAs in the E-TABM-282 dataset and confirmed differential expression of five lncRNAs (fold change > 2, *P* < 0.05): ENSG00000247225, ENSG00000241732, ENSG00000177133, ENSG00000244020, and TCL6. Because ENSG00000247225 and ENSG00000241732 had been removed from Ensembl, we investigated ENSG00000177133, ENSG00000244020, and TCL6 in this study (Table 2). These three lncRNAs were downregulated in ccRCC samples.

**Table 2:** lncRNAs differentially expressed between ccRCC samples and normal renal tissues both in GSE53757 and E-TABM-282

Probe set ID	Symbol	GSE53757		E-TABM-282		HGNC symbol	Description
		Fold change	Adjusted *P* value	Fold change	Adjusted *P* value		
231275_at	ENSG00000177133	0.33	< 1E–07	0.48	0.014	LINC00982	long intergenic non-protein coding RNA 982
211456_x_at	ENSG00000244020	0.25	< 1E–07	0.47	0.038	MT1HL1	metallothionein 1H-like 1
219840_s_at	TCL6	0.22	< 1E–07	0.47	0.044	TCL6	T-cell leukemia/lymphoma 6

HGNC: Human Genome Nomenclature Committee

### Validation of the expression profiles using RT-PCR

**Figure 3:**
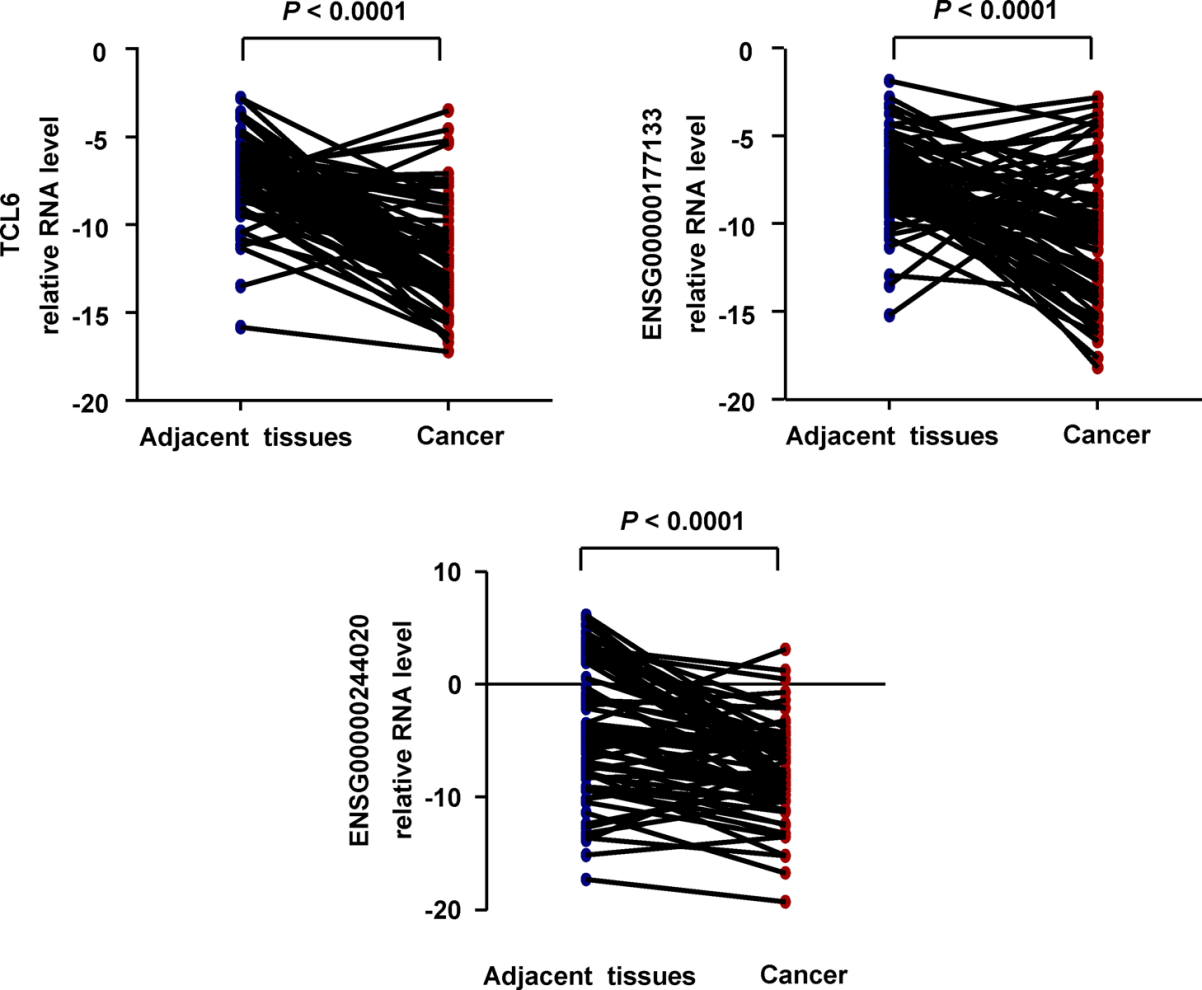
Quantification of the expression of three candidate lncRNAs in ccRCC and paired adjacent tissue (n = 71; Non-parametric Mann-Whitney test)

To validate the microarray data, we examined the expression of these three lncRNAs in 71 ccRCC and adjacent normal renal tissue samples. Decreased expression of three lncRNAs (ENSG00000177133, TCL6, and ENSG00000244020) was observed in ccRCC compared to adjacent normal tissue (*P* < 0.05) (Figure 3).

### The clinical relevance of lncRNAs in ccRCC

**Figure 4:**
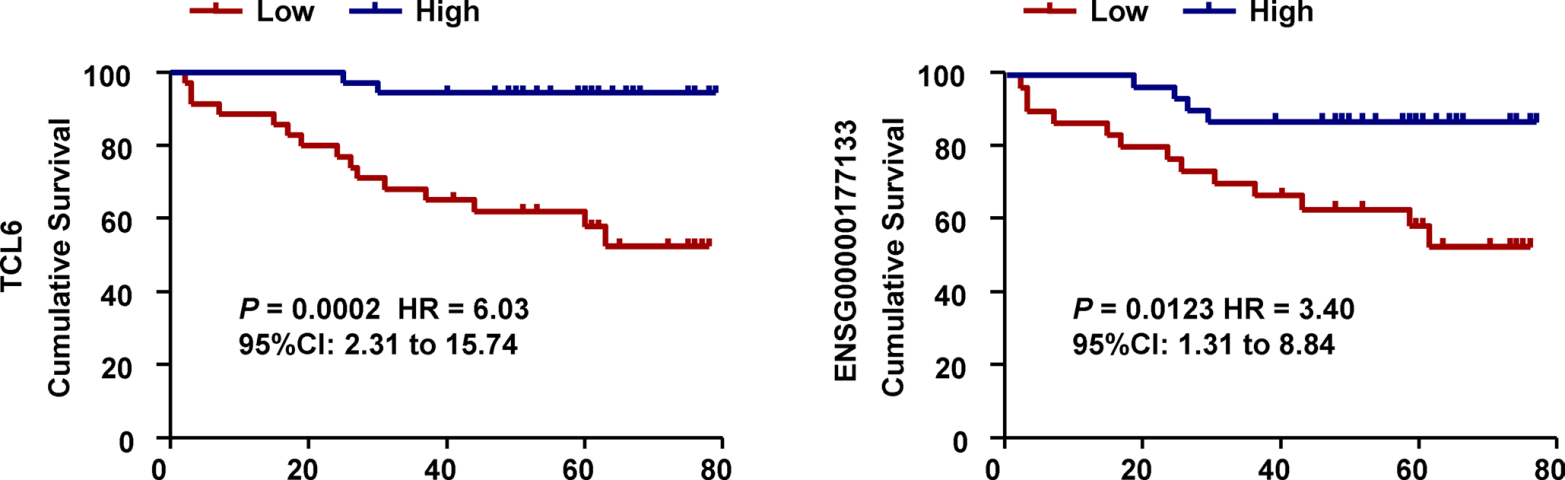
Associations between lncRNAs and survival in 71 patients with ccRCC Survival analysis indicates that patients with tumors that have high TCL6 or ENSG00000177133 expression have a more favorable prognosis compared to those with lower expression (Log-rank test).

**Figure 5:**
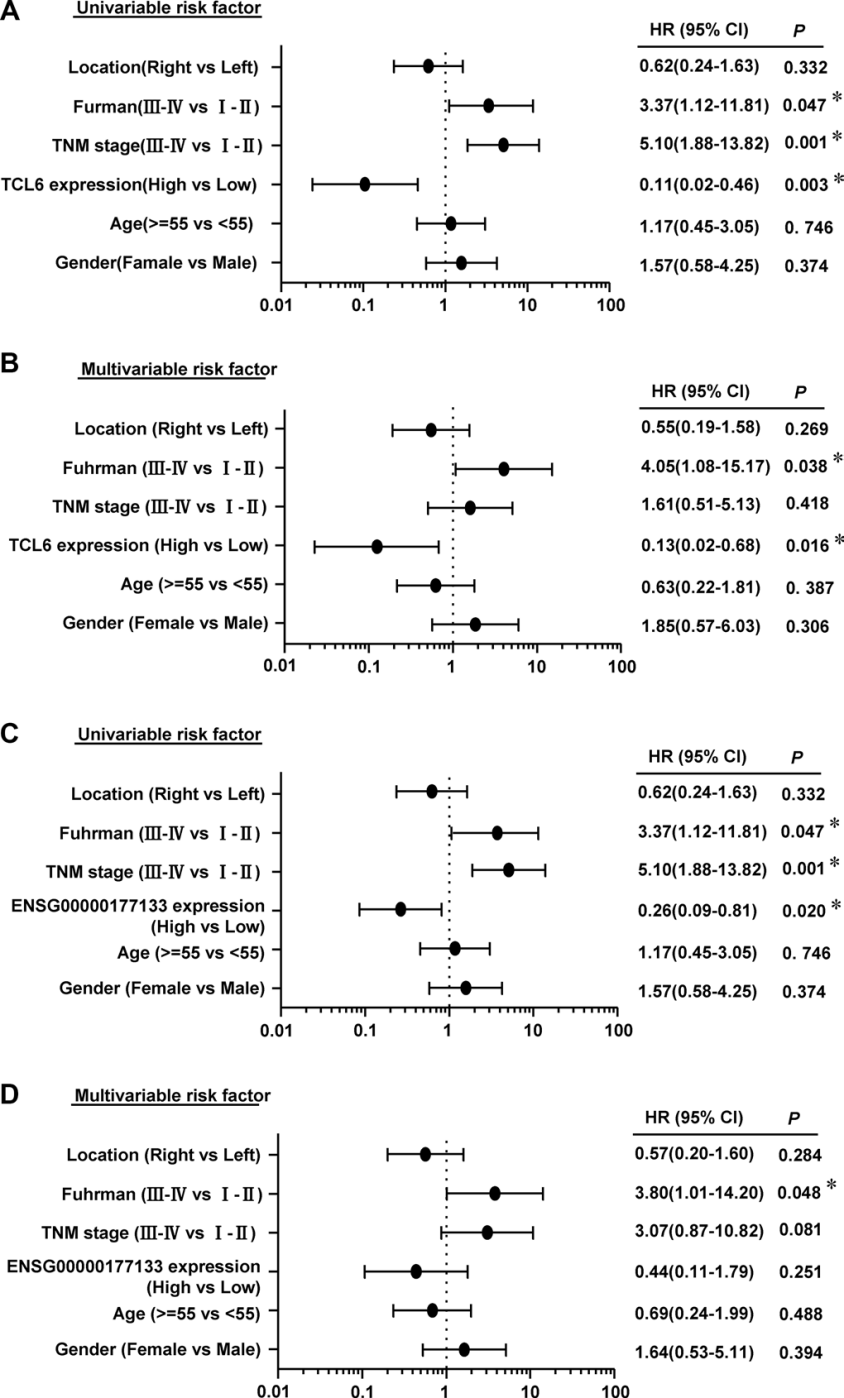
Univariate (A) and multivariate analyses (B) of TCL6 in ccRCC patients Univariate (**C**) and multivariate analyses (**D**) of ENSG00000177133. All bars correspond to 95% CIs.

Kaplan-Meier analysis indicated that reduced expression of TCL6 or ENSG00000177133 was associated with poor prognosis (Figure 4). In contrast, ENSG00000244020 had no predictive value for prognosis. Univariate (Figure 5A) and multivariate (Figure 5B) regression analyses demonstrated that TCL6 expression was an independent predictor of ccRCC aggressiveness and had significant hazard ratios [HRs] for predicting clinical outcome. Although high expression of ENSG00000177133 was associated with a decreased risk of cancer-related death in univariate regression analysis (*P* = 0.02, HR = 0.26, 95% confidence interval [CI]: 0.09–0.81, Figure 5C), a multivariate model indicated that ENSG00000177133 could not predict prognosis after adjustment for potential confounding factors (*P* = 0.251, HR = 0.44, 95% CI: 0.11–1.79, Figure 5D).

We next analyzed the association between TCL6 expression and clinicopathological features of ccRCC patients. TCL6 expression was negatively correlated with T stage, N stage, M stage, and TNM stage. No correlation was observed between TCL6 expression and other clinicopathological features (Table 3).

**Table 3:** Correlations between TCL6 expression and clinicopathological features

	TCL6		
Clinicopathological feature	Low (*n*= 35)	High (*n*= 36)	*P* value
**Age(years)**			0.192
<55	15	21	
≥55	20	15	
**Gender**			0.734
Male	25	27	
Female	10	9	
**Tumor location**			0.904
Left	17	18	
Right	18	18	
**TNM stage**			0.001*
Stage I-–II	14	35	
Stage III–IV	21	1	
**T classification**			0.001*
T1, 2	18	35	
T3, 4	17	1	
**Lymph node metastasis**			0.005*
N0	28	36	
N1	7	0	
**Distant metastasis**			
M0	29	36	0.011*
M1	6	0	
**Fuhrman Grade**			
Stage I–II	12	15	0.522
Stage III–IV	23	21	

### The functional role of TCL6 as a tumor suppressor

**Figure 6:**
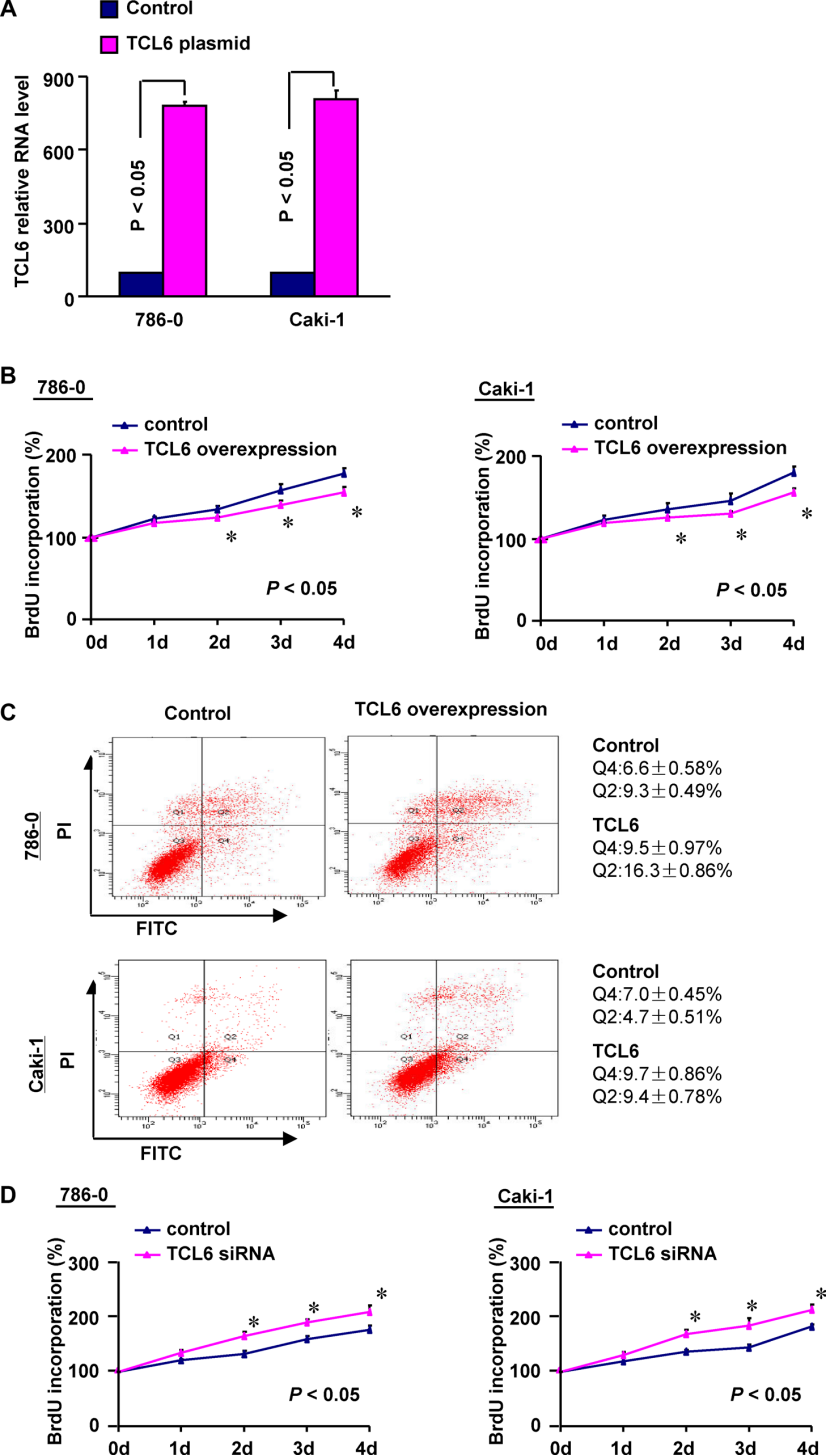
(**A**) Quantitative RT-PCR confirming ectopic expression of TCL6 in ccRCC cell lines (786-0 and Caki-1) after TCL6 plasmid transfection (*n* = 3; *P* < 0.05; Non-parametric Mann-Whitney test). Error bars represent the SD. (B) TCL6 overexpression inhibits renal cell growth *in vitro* (*n* = 3; Non-parametric Mann-Whitney test). (**C**) Flow cytometry analysis of apoptosis after TCL6 overexpression in renal cells (*n* = 3). (**D**) Cell proliferation assays after TCL6 knockdown with siRNA (*n* = 3; Non-parametric Mann-Whitney test).

To elucidate the functions of TCL6 in ccRCC pathogenesis, we overexpressed TCL6 in two ccRCC cell lines (786–0 and Caki-1). We first confirmed that TCL6 was overexpressed in both cell lines (Figure 6A). Overexpression of TCL6 inhibited ccRCC cell proliferation and promoted apoptosis (Figure 6B and 6C). Conversely, knockdown of TCL6 promoted proliferation (Figure 6D). These data suggest that TCL6 may function as a tumor suppressor in ccRCC.

## DISCUSSION

Previous studies have demonstrated that lncRNAs regulate tumorigenesis and may provide new strategies for diagnosis and treatment [10, 11]. There have been conflicting reports on the functions of lncRNAs in ccRCC [12–15]. Several lncRNAs have been associated with advanced clinicopathological features and poor prognosis. These lncRNAs (e.g. CADM1-AS1 and NBAT-1) may function as tumor suppressors in ccRCC [12, 13]. Conversely, other lncRNAs such as RCCRT1 and MALAT1 have been shown to promote ccRCC cell migration and invasion [14, 15]. Here, we investigated lncRNA expression in ccRCC by re-annotating the HG-U133 Plus 2.0 probe sets from the GEO database. The clinical potential and prognostic significance of the lncRNAs was assessed in ccRCC.

Differential expression of several lncRNAs was observed between ccRCC and adjacent normal renal tissue. According to the microarray results from two GEO databases (GSE-53757 and E-TABM-282), five lncRNAs were differentially expressed in ccRCC samples compared to matched counterparts. The real time-PCR results for 71 pairs of ccRCC samples confirmed the variable expression of three lncRNAs in ccRCC. Therefore, these lncRNAs could potentially be used as diagnostic biomarkers in ccRCC.

Several studies have explored the association between lncRNA expression and the prognosis of ccRCC patients. However, the results differed depending on the lncRNA. Blondeau et al. identified novel lncRNAs that were dysregulated in ccRCC, but found no correlation between lncRNA expression and patient clinicopathological features [16]. Other studies provided evidence that lncRNAs were involved in renal carcinogenesis and were correlated with ccRCC patient prognosis [17, 18]. In this study, the expression of TCL6 and ENSG00000177133 was correlated with ccRCC patient prognosis. Univariate and multivariate regression analyses showed that TCL6 expression was an independent predictor of ccRCC aggressiveness and was negatively correlated with the T, N, M, and TNM stage. Previous study have found that TCL6 (located on chromosome 14, NC_000014.9) was a candidate gene potentially involved in leukemogenesis [19]. Our data indicate that TCL6 is a prognostic marker for ccRCC.

Decreased expression of TCL6 was associated with advanced clinicopathological features in ccRCC patients and poor prognosis. We hypothesized that TCL6 could inhibit the growth of ccRCC cells and promote apoptosis. To test this hypothesis, we overexpressed TCL6 in 786–0 and Caki-1 cells. Transfection of 786–0 and Caki-1 cells with TCL6 resulted in a decrease in cell proliferation and increase in apoptosis. These results suggested that TCL6 might act as a tumor suppressor in ccRCC. Additional studies are required in order to elucidate the mechanisms underlying the effects of TCL6 on ccRCC cells.

A major strength of the present study is that the data were derived from two separate cohorts. We selected the most significantly altered lncRNAs and validated the expression in our clinical samples. Our study was limited in that the HG-U133 Plus 2.0 arrays represented only a fraction of all possible lncRNAs. Therefore, the lncRNAs we identified may not represent the complete lncRNA population that is involved in ccRCC pathogenesis. Additionally, the sample size of the E-TABM-282 dataset was relatively small, and therefore further studies with larger sample sizes are required to confirm our results.

In summary, we have identified multiple novel lncRNAs that are differentially expressed in ccRCC and found that reduced TCL6 expression was associated with poor prognosis in ccRCC patients. These findings may facilitate ccRCC diagnosis, classification, prognosis, and evaluation of new therapeutic strategies.

## MATERIALS AND METHODS

### Patients and samples

The primary tumor samples and adjacent normal tissue were collected from 71 patients at Fudan University Shanghai Cancer Center (FUSCC) who underwent nephrectomy or partial nephrectomy for ccRCC. None of the patients received preoperative radio-chemotherapy. Patients received regular follow-up through phone calls or clinic visits every 3 months until December 2014 or until the date of death. Overall survival (OS) was defined as the time from the date of surgery to the date of death or final clinical follow-up. The study protocol was approved by the Ethics Committee of FUSCC and patients provided written informed consent prior to enrollment.

### GEO gene expression data

Two ccRCC gene expression datasets were included in our study: GSE-53757 (72 ccRCC and 72 normal patients) and E-TABM-282 (16 ccRCC and 11 normal patients) [8, 9]. The raw CEL files for these two datasets on the Affymetrix HG-U133 Plus 2.0 platform were downloaded from GEO and ArrayExpress database. The lncRNA expression profiles were highlighted based on the NetAffx annotation of the probe sets as well as the Refseq and Ensembl annotations for the lncRNAs as described previously [20]. These datasets corresponded to all available public datasets that fulfilled the following criteria: (i) a comparison between ccRCC and normal kidney tissue was performed; (ii) the same chip platform (Affymetrix HG-U133 Plus 2.0 Array) was used in the analysis; (iii) the dataset contained more than three samples that met the quality control standard in the experimental and control groups. Stringent filtering criteria (fold change > 2, *P* < 0.05) was used in our analysis.

The heatmap was generated by MultiExperiment Viewer (MeV, version 4.6) clustering with Euclidean distance based on the data downloaded from Geo database GSE53757.

### RNA isolation and RT-PCR

Tumor tissue samples (collected and preserved for subsequent RNA analysis) were homogenized using an electric homogenizer, and the total RNA extracted using the TRIzol reagent. Next, 1 μg of total RNA was reverse-transcribed using the PrimeScriptPTMP RT Reagent Kit (Perfect Real Time; Takara, Shiga, Japan). The RNA levels were measured using a real-time quantitative PCR system. The amplified transcript level of each specific gene was normalized to GAPDH expression. The primers (Table 4) were provided by Sheng Gong Company (Shanghai, China).

**Table 4:** Primer sequences of three candidate lncRNAs and GAPDH

Gene symbol	Forward primer	Reverse primer
ENSG00000177133	5′- CTGTTGTGGGTGCTGTGATT-3′	5′- GTCAGGGTGCTCTTCCTCTG-3′
ENSG00000244020	5′- TTCTCTTCTCGCTTGGGAAC-3′	5′- CTTGCAGGAGGTGCATTTG-3′
TCL6	5′- TGTCTCATTCGCCTCTGGAT-3′	5′- GTCTCCCTCCTTCTGCCTTT-3′
GAPDH	5′- GCATTGCCCTCAACGACCAC -3′	5′-CCACCACCCTGTTGCTGTAG-3′

### Cell culture and treatment

Human ccRCC cell lines (786-0 and Caki-1) were cultured in RPMI-1640 medium (Gibco, Carlsbad, CA, USA) supplemented with 10% fetal bovine serum at 37°C in a 5% CO_2_ atmosphere. Transfection of the TCL6 and non-specific negative control plasmids was performed using the FuGENE transfection reagent (Life Technologies, Shanghai, China). The siRNA against TCL6 was transfected (50 nM) using the DharmaFECT 1 siRNA transfection reagent (Thermo Scientific Dharmacon Inc., Lafayette, CO, USA), while nonspecific siRNA was used as a negative control.

### Cell proliferation assay

Cell proliferation was assessed using the BrdU incorporation assay (Roche, Mannheim, Germany). Briefly, cells were seeded into 96-well plates at an initial density of 4 × 10^3^ cells/well. BrdU labeling solution (10 μL/well) was added to the cells at the indicated time points. After a 2 h incubation, the culture medium was removed and the cells fixed. The DNA was denatured with FixDenat (200 μL/well) and anti-BrdU-POD working solution (100 μL/well) was then added to the cells and incubated for 90 min. Immune complexes were detected by the subsequent substrate reaction. The reaction product was quantified by measuring the absorbance at 370 nm (reference wavelength: approximately 492 nm).

### Flow cytometry analysis of apoptosis

Flow cytometry was performed to analyze apoptosis. An annexin-V fluorescein isothiocyanate (FITC)/propidium iodide (PI)_double stain assay (Biovision Inc, Mountain View, CA, USA) was performed using the manufacturer's protocol. Both floating and trypsinized adherent cells were collected, resuspended in 500 μL of binding buffer containing 2.5 μL of annexin-V FITC and 5 μL of PI, and then incubated for 5 min in the dark at room temperature before analysis by flow cytometry.

### Statistical analysis

Statistical analyses were performed using the SPSS software (SPSS 17.0.1, Inc., Chicago, IL, USA). Data from atleast three independent experiments performed in triplicate are presented as the mean ± standard deviation (SD). Kaplan Meier estimates were used to analyze the impact of lncRNA expression (stratified according median expression levels) on patient survival. Univariate and multivariate Cox regression analyses were performed to examine the effect of lncRNA expression on survival. Enumeration data were analyzed using Chi-squared or Fisher's exact tests. Statistical tests and *P*-values were two-sided. A *P*-value < 0.05 was considered statistically significant.
